# Green Synthesis and Characterization of Bismuth Oxide Nanoparticle Using *Mentha Pulegium* Extract

**DOI:** 10.22037/ijpr.2019.15578.13190

**Published:** 2020

**Authors:** Negar Motakef-Kazemi, Masoumeh Yaqoubi

**Affiliations:** a *Department of Medical Nanotechnology, Faculty of Advanced Sciences and Technology, Tehran Medical Sciences, Islamic Azad University, Tehran, Iran. *; b *Department of Nanochemistry, Faculty of Pharmaceutical Chemistry, Tehran Medical Sciences, Islamic Azad University, Tehran, Iran.*

**Keywords:** Green synthesis, Bismuth oxide, Nnanoparticles, Mentha pulegium, Extract

## Abstract

In recent years, green synthesis of nanoparticles has attracted a great attention because of medicine and biological applications. In this work, bismuth oxide nanoparticles (Bi_2_O_3_ NP) was prepared via green synthesis using *mentha pulegium *aqueous extract after 24 h at 90°C. The product was characterized by ultraviolet-visible (UV-VIS) spectrophotometer, Fourier transform infrared (FTIR), X-ray diffraction (XRD), dynamic light scattering (DLS), scanning electron microscopy (SEM), transmission electron microscope (TEM), energy-dispersive X-ray spectroscopy (EDS), and diffuse reflection spectroscopy (DRS). The antibacterial activities of the samples were determined against *Salmonella *and *Escherichia coli* (*E.Coli*) as Gram-negative bacterial and *Staphylococcus aureus* (*S.aureus*) as Gram-positive bacterial. The objectives of this study were the green synthesis of bismuth oxide nanoparticles using aqueous extract with a good potential for UV blocking and antibacterial activity. Based on the obtained results, Bi_2_O_3_ NPs can have a good candidate for different applications.

## Introduction

Today, nanotechnology has attracted a great attention in different fields. The nanoparticles have nanoscale dimensions in a range of 1–100 nm with unique properties due to small size and high surface area (1). Metal oxide nanoparticles have received a research attention in many areas with unique and wide-ranging physicochemical properties (2). Bismuth oxide nanoparticle is a good candidate of metal oxide for various applications in preparation of nanostructures (3), photocatalyst (4, 5), solid oxide fuel cell (6), gas sensor (7) catalyst for oxidation of hydrocarbons (8), catalytic performance for reduction (9), water purification (10), photovoltaic (11), biomedical (12, 13), and antibacterial effect (14). Various polymorphs of bismuth oxide have included α-Bi_2_O_3_, β-Bi_2_O_3_, γ-Bi_2_O_3_, δ-Bi_2_O_3_, ε-Bi_2_O_3_, and ω-Bi_2_O_3_ based on temperature. The stable polymorph is monoclinic α-Bi_2_O_3_ in low temperature and cubic δ-Bi_2_O_3_ in high temperature (15, 16). The increase of temperature caused the decrease of tetragonal β-Bi_2_O_3_ structure and the show monoclinic α-Bi_2_O_3_ in XRD patterns (17). 

Bismuth nanostructures can be fabricated by several methods such as solution (18, 19), solution combustion (20), solvothermal (21), hydrothermal (22), laser ablation (23), microwave (24), sol–gel (25), flame spray pyrolysis (26), thermal decomposition (27, 29), electrodeposition (30), thermal oxidation (31), chemical vapour deposition (32), and green synthesis (33). The green synthesis is a challenge for preparation of monodispersed nanoparticles with specific sizes and shapes (34). Biosynthesis methods have more advantages than other classical synthesis procedures due to the easy availability, rich biodiversity, and eco-friendly processes (35, 36). Green synthesis is very easy and cost-effective method for production of nanoparticles using the extract. The plant extract can act as reducing and capping agent for the reduction of metal ions and the formation of nanoparticles because of presence of the various biomolecules such as flavonoids, enzymes, proteins, phenolic acid, alkaloids, and terpenoids (37, 38). Silver nanoparticles were synthesized by *Mentha pulegium* (pennyroyal) leaf extract for antibacterial application (39, 40). 

The ultraviolet light can be caused the increase of risk for skin cancer and ocular damage. The UV radiation included three regions UV-A (320–400 nm), UV-B (280-320 nm), and UV-C (180-280 nm). The earth’s atmosphere traps all UV-C and more than 99% of UV-B radiation. The UV-A blocking is the most important consideration for hazard prevention of exposure to direct sunlight (41). Recently, bismuth oxide was reported as the UV-absorber (16). UV blocking ability is different in nanomaterial compared with bulk material because of small size and large surface area to volume ratio (42, 43). Antibacterial activity is another good application of bismuth oxide nanoparticles against some pathogenic Gram-negative bacteria (14). In the present study, we have developed a facile green synthesis method for preparation of bismuth oxide nanoparticles using *mentha pulegium* extract. The objective of this research was to achieve the goals of green synthesis of Bi_2_O_3_ NPs for potential application as UV blocking and antibacterial activity.

## Experimental

*Materials*

All chemicals were analytical grade. Double distilled (DD) water was used in all experiments. Bismuth nitrate (Bi(NO_3_)_3_) as a bismuth precursor was purchased from Merck. The fresh leaves of *mentha pulegium* were prepared to make aqueous extract as reducing agent for the green synthesis of bismuth oxide nanoparticles from Siahkal region of Guilan province, Iran. 

*Green synthesis of bismuth oxide nanoparticles*

*Mentha pulegium* also named pennyroyal is a species of flowering plant in the mint family. Firstly, *mentha pulegium* leaves were gathered in May from Siahkal region of Guilan province. The leaves were washed thoroughly with double distilled water to remove the dust particles. Then, they were approved by Herbarium (1634-AUPF) Islamic Azad University of Tehran Medical Sciences. Finally, the leaves cut into the very fine pieces and dried in the presence of sunlight. For the preparation of leaf extract, 20.0 g of the leaves of *mentha pulegium* was immersed in the 200 mL of DD water and boiled at 90 °C for 2 h. The obtained leaf extract was kept for cooling at room temperature, filtered using Whatman filter papers. For the green synthesis bismuth oxide nanoparticles, the amount of 2 g bismuth nitrate was solved in 10 mL DD water at 90 °C and mixed with 20 mL of *mentha pulegium* aqueous extract at 90 °C under constant stirring. Based on the UV-Visible spectrophotometer results, bismuth oxide NPs were prepared after 24 h at 90°C. The Schematic reaction is as follows:

2BiNO_3_ + Pennyroyal extract > Bi_2_O_3_

Then, the result samples were washed several times with DD water, and dried in a vacuum. The product was heated at 550 °C in a furnace and static atmosphere of air for 5 h to ensure the removal of impurities. 

*Characterization*

The optimum time of synthesized nanoparticles was analyzed by UV-Visible spectrophotometer (Shimadzu, UV-1650PC, and Japan) for solution sample. Fourier transform infrared spectrum was recorded on a Unicam Matson 1000 FT-IR spectrophotometer using a KBr disks at room temperature. Powder X-ray diffraction pattern was performed for evaluation of crystalline structure of bismuth oxide NP using a Philips Company X’pert diffractometer utilizing Cu-Ka radiation (ASENWARE, AW-XBN300, China). Size and size distribution of nanoparticles were investigated by dynamic light scattering (ZEN314, England). Scanning electron microscope was employed to observe the morphology and size of nanoparticles (KYKY, EM3200, and China). Also, morphology and size of nanoparticles were evaluated by transmission electron microscope (Zeiss-EM10C-100 KV, Germany). The energy-dispersive X-ray spectroscopy evaluated the elemental and chemical analysis of bismuth oxide NP (ASK SEM-CL View VIS, Oxford instruments, UK). Diffuse reflection spectroscopy investigated the UV protective properties of nanoparticles (Shimadzu, UV2550, and Japan) for solid sample. The antibacterial activities were evaluated by disk diffusion method against Salmonella (strains ATCC 1231) and *E.Coli *(strains ATCC 25922) as Gram-negative bacteria and S.aureus (strains ATCC 6538) as Gram-positive bacteria for bismuth oxide nanoparticles.

## Results and Discussion

*UV-VIS*

**Figure 1:**
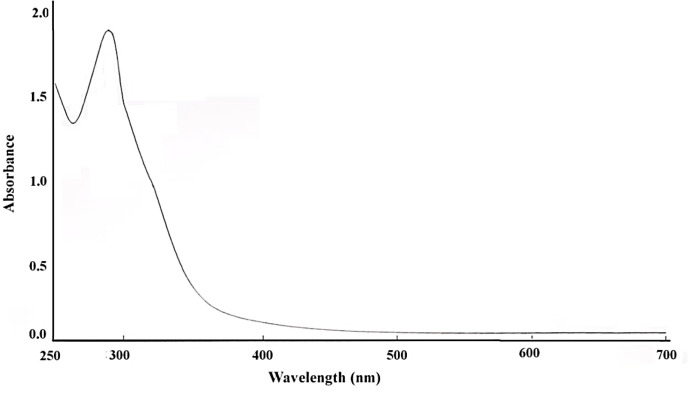
UV-VIS spectrum of bismuth oxide nanoparticle

UV-Visible spectroscopy confirmed the presence of nanoparticles by reduction of bismuth ions in the solution (Figure 1). The bismuth oxide nanoparticles were placed in a quartz cuvette and observed for wavelength scanning between 250 to 700 nm with distilled water as a reference. The absorption peak was observed at 290 nm, which is characteristic bismuth. UV-Visible spectroscopy is similar to the previous report (44).

*FTIR*

**Figure 2:**
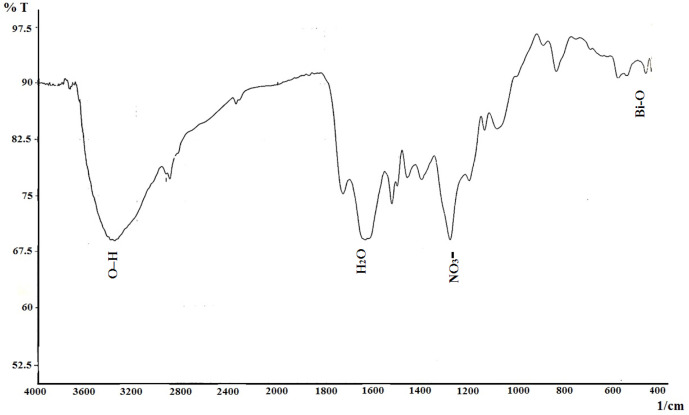
FTIR bismuth oxide nanoparticle

The FTIR absorption spectrum was recorded in the range of 400–4000 cm^-1^ (Figure 2) to determine functional groups and qualitative formation of bismuth nanoparticles. The O–H stretching vibrations appeared at 3363~3414 cm^-1^. The C-O vibrations attributed at 2330 cm^-1 ^corresponding to CO_2 _of environment. The peak was observed at 1629 cm^-1 ^corresponding to H_2_O. The peak at 1261 cm^-1^ is related to nitrate (NO_3_^-^) group. The peak at 542 cm^-1^ is originated from the metal-oxygen (Bi-O) vibration. Fourier transform infrared result is similar to the previous report (25). 

*XRD*

**Figure 3:**
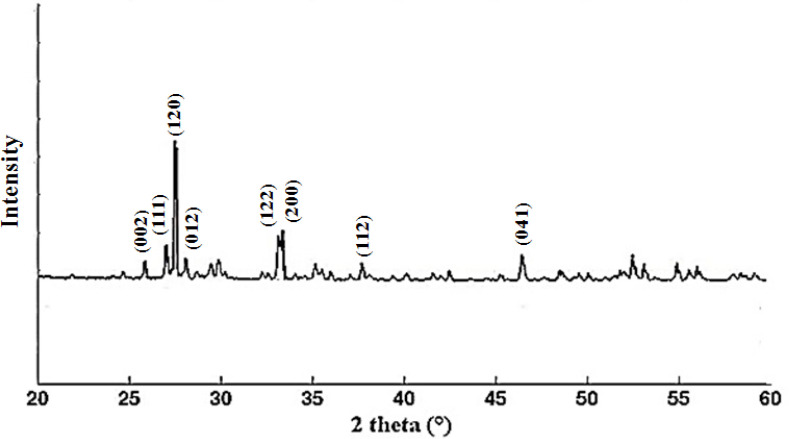
XRD bismuth oxide nanoparticle

X-ray diffraction measurement was used to determine the crystalline structure of bismuth nanoparticles in 2θ range 20 to 60° after decomposition at 550 °C (Figure 3). The sharp peak was observed at 2θ around 28°, and indexed the monoclinic α-Bi_2_O_3_ for all diffraction peaks (JCPDS card No. 41-1449). This result is similar to the previously reported pattern (5). 

*DLS*

**Figure 4:**
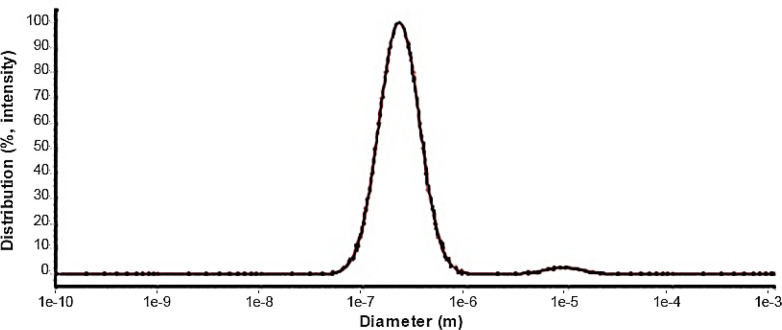
DLS bismuth oxide nanoparticle

The dynamic light scattering was used to find out the size and distribution diagram of nanoparticles (Figure 4). DLS results showed a single-peak with size of about 220 nm and a narrow distribution at room temperature and confirmed the SEM result.

*SEM*

**Figure 5:**
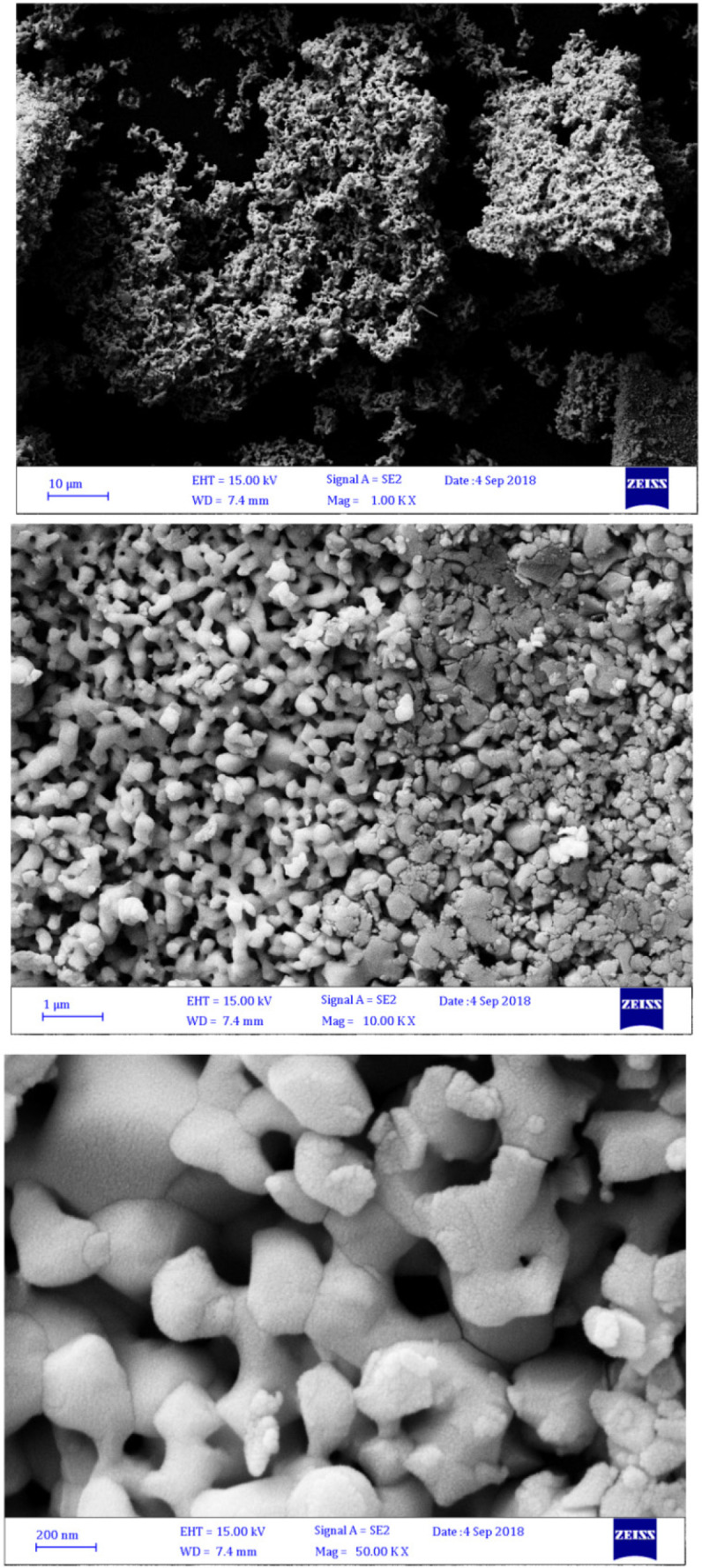
SEM bismuth oxide nanoparticle

Morphology and size of bismuth oxide nanoparticles were characterized by scanning electron microscope (Figure 5). The SEM image demonstrated 200 nm for particle size, and confirmed the DLS result. 

*TEM*

**Figure 6:**
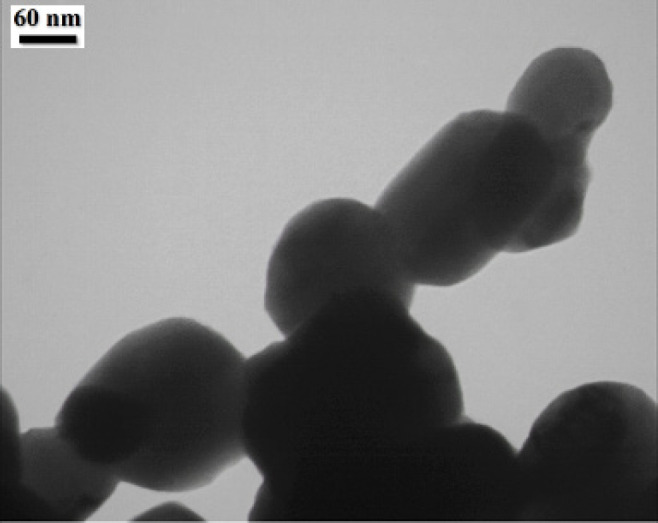
TEM bismuth oxide nanoparticle

Transmission electron microscope was employed to observe morphology and size of bismuth oxide nanoparticles (Figure 6). The TEM image demonstrated about 120 nm for particle size. 

*EDS*

**Figure 7:**
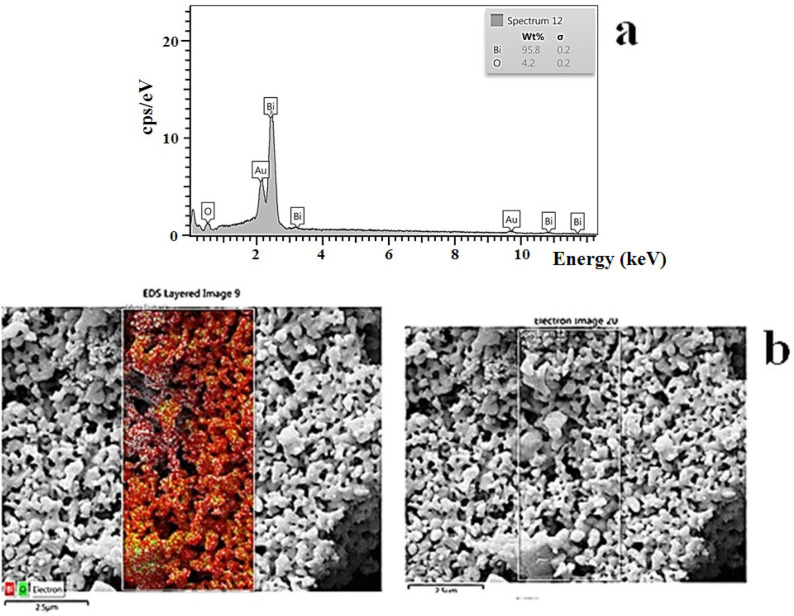
aEDS, and b elemental map image of bismuth oxide nanoparticle

The energy-dispersive X-ray spectroscopy was used to evaluate the chemical composition of bismuth oxide nanoparticles. This analysis clearly showed the identification strong peaks of bismuth (Bi) and oxygen (O) elements. The EDS analysis of bismuth oxide NP exhibited absorption bands with peaks at 2.4, 3.2, 10.8, and 11.8 keV, which illustrated a typical absorption of the metallic bismuth. The energy-dispersive X-ray spectroscopy and mapping of bismuth oxide nanoparticles were carried out for elemental analysis (Figure 7).

*DRS*

**Figure 8:**
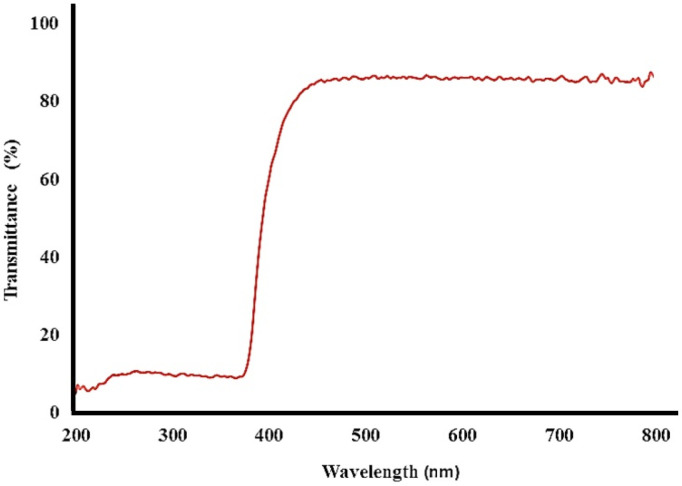
DRS bismuth oxide nanoparticle

The DRS absorption spectrum of bismuth oxide nanoparticles showed the ultraviolet protective properties in three Ultraviolet: UV-A, UV-B, and UV-C (Figure 8). The absorption peak was observed in range of 200-400 nm, approving the UV protective property of the nanoparticles. Based on DRS spectra of product, bismuth oxide nanoparticles affected the light absorption property and absorbed 99% ultraviolet. 

*Antibacterial activity*

Antibacterial activity was measured against Gram-negative and Gram-positive bacterial for different concentrations of the samples by determination of minimum inhibitory concentration (MIC), minimum bactericidal concentration (MBC), and zone inhibition. Table 1 was presented the results of MIC and MBC, and Table 2 was showed the results of zone inhibition. 

Initially, fresh bacteria were prepared in the growth medium of Mueller Hinton Broth. Then, some of the bacteria were dissolved in sterile physiological serum to obtain an opacity equal to OD=0.1 (half McFarland). On the other hand, different concentrations of the samples were sterilized using Muller Hinton Broth. Finally, a volume of bacteria was added to each of the samples in physiological serum to give the count of 100,000 bacteria per mL and placed in an incubator at 37 °C. The positive control group (growth medium with bacteria) and the negative control group (growth medium and samples) are also considered. After 24 h, bacterium darkness was assessed. MIC determination was recorded by the samples without bacterium darkness. MBC determination was considered for of Non-darkness samples without bacterium grown in Muller Hinton agar medium. Disk diffusion method was done for measurement of zone inhibition after determination of MIC and MBC in three concentrations (including 10, 5, 2,5 mg/mL).

First, blank discs were immersed in growth medium containing each concentration for 5 min. Then, fresh growth medium (with OD=0.1 in sterile physiology serum) was cultured with sterile swabs in Muller Hinton agar medium. The discs were stained with different concentrations of samples at appropriate distances in agar medium. Finally, the discs were incubated in a 37 °C incubator for 24 h. The diameter of zone inhibition was measured by the ruler. According to the results, Salmonella as Gram-negative bacterial is a good candidate for antibacterial activity of bismuth oxide nanoparticles. Future prospect of green synthesis can have huge application for nanomaterial in the field of food, pharmaceutical, and cosmetic industries and thus become a major area of research. 

**Table 1:** MIC and MBC of bismuth oxide nanoparticle

Sample	*E.Coli* (ATCC 25922 )		*S.aureus* (ATCC 6538 )		*Salmonella* (ATCC 1231)	
	MIC	MBC	MIC	MBC	MIC	MBC
Bi_2_O_3_	10 mg/mL	10 mg/mL	10 mg/mL	10 mg/mL	2.5 mg/mL	10 mg/mL

**Table 2:** The zone inhibition of bismuth oxide nanoparticle

**Concentration (mg/mL)**	**Zone inhibition (mm)**		
	***E.Coli*** ** (ATCC 25922) **	***S.aureus*** ** (ATCC 6538)**	***Salmonella*** ** (ATCC 1231)**
10	2	1	13.6
5	1	0	0
2.5	0	0	0

## Conclusion

The bismuth oxide nanoparticles were prepared by green method using *mentha pulegium *aqueous extract as a reducing agent. The XRD spectrum confirmed the monoclinic α-Bi_2_O_3 _crystalline structure. The DLS, SEM and TEM results showed the size in nanometer scale with narrow distribution and the particle size was estimated 150 nm. In the present study, we successfully observed UV blocking and antibacterial activity applications of bismuth oxide NPs. These properties can be resulted in many advantages in the future with less harm and toxicity to the human health and more safety. 

## References

[1] Tabrez S, Musarrat J, Al-khedhairy AA (2016). Colloids and surfaces B: biointerfaces countering drug resistance, infectious diseases, and sepsis using metal and metal oxides nanoparticles: current status. Colloids Surf B Biointerfaces..

[2] Falcaro P, Ricco R, Yazdi A, Imaz I, Furukawa S, Maspochb D, Ameloot R, Evans JD, Doonan CJ (2016). Application of metal and metal oxide nanoparticles@MOFs. Coord. Chem. Rev..

[3] Fan HT, Pan SS, Teng XM, Ye C, Li GH, Zhang LD (2006). δ-Bi2O3 thin films prepared by reactive sputtering: Fabrication and characterization. Thin Solid Films.

[4] Li R, Chen W, Kobayashi H, Ma C (2010). Platinum- nanoparticle-loaded bismuth oxide: an efficient plasmonic photocatalyst active under visible light. Green Chem..

[5] Raza W, Haque MM, Muneer M, Harada T, Matsumura M (2015). Synthesis, characterization and photocatalytic performance of visible light induced bismuth oxide nanoparticle. J. Alloys. Compd.

[6] Gong Y, Ji W, Zhang L, Xie B, Wang H (2011). Performance of (La,Sr)MnO3 cathode based solid oxide fuel cells: effect of bismuth oxide sintering aid in silver paste cathode current collector. J. Power Sources.

[7] Gou X, Li R, Wang G, Chen Z, Wexler D (2009). Room-temperature solution synthesis of Bi2O3 nanowires for gas sensing application. Nanotechnology.

[8] Malik P, Chakraborty D (2010). Bi2O3-Catalyzed Oxidation of Aldehydes with t-BuOOH. Tetrahedron Lett..

[9] Xia F, Xu X, Li X, Zhang L, Zhang L, Qiu H, Wang W, Liu Y, Gao J (2014). Preparation of bismuth nanoparticles in aqueous solution andi catalytic performance for the reduction of 4nitrophenol. Ind. Eng. Chem. Res..

[10] Schlesinger M, Weber M, Schulze S, Hietschold M, Mehring M (2013). Metastable β-Bi2O3 nanoparticles with potential for photocatalytic water purification using visible light irradiation. Chemistry.

[11] Mahmouda WE, Al-Ghamdia AA (2011). Synthesis and properties of bismuth oxide nanoshell coated polyaniline nanoparticles for promising photovoltaic properties. Polym. Adv. Technol..

[12] Oviedo MJ, Contreras OE, Rosenstein Y, Vazquez-Duhalt R, Macedo ZS, Carbajal-Arizaga GG, Hirata GA (2016). New bismuth germanate oxide nanoparticle material for biolabel applications in medicine. J. Nanomater..

[13] Abudayyak M, Oztas E, Arici M, Ozhan G (2017). Investigation of the toxicity of bismuth oxide nanoparticles in various cell lines. Chemosphere.

[14] Jassim AMN, Farhan SA, Salman JAS, Khalaf KJ, Al Marjani MF, Mohammed MT (2015). Study the antibacterial effect of bismuth oxide and tellurium nanoparticles. Int. J. Chem. Biol. Sci..

[15] Mehring M (2007). From molecules to bismuth oxide-based materials: Potential homo- and heterometallic precursors and model compounds. Coord. Chem. Rev.

[16] Perez-Mezcua D, Sirera R, Jimenez R, Bretos I, De Dobbelaere C, Hardy A, Baelc MKV, Lourdes Calzada M (2014). A UV-absorber bismuth (III)-Nmethyldiethanolamine complex as a lowtemperature precursor for bismuth-based oxide thin films. J Mater. Chem. C..

[17] Hou J, Yang C, Wang Z, Zhou W, Jiao S, Zhu H (2013). In situ synthesis of α-β-phase heterojunction on Bi2O3 nanowireswith exceptional visible-light photocatalytic performance. Appl. Catal. B..

[18] Solanki PR, Singh J, Rupavali B, Tiwari S, Malhotr BD (2017). Bismuth oxide nanorods based immunosensor for mycotoxin detection. Mater. Sci. Eng. C..

[19] Xia F, Xu X, Li X, Zhang L, Zhang L, Qiu H, Wang W, Liu Y, Gao J (2014). Preparation of bismuth nanoparticles in aqueous solution and its catalytic performance for the reduction of 4-Nitrophenol. Ind. Eng. Chem. Res..

[20] La J, Huang Y, Luo G, Lai J, Liu C, Chu G (2012). Synthesis of bismuth oxide nanoparticles by solution combustion method. Particul. Sci. Technol..

[21] Wu J, Qin F, Lu Z, Yang HJ, Chen R (2011). Solvothermal synthesis of uniform bismuth nanospheres using poly(N-vinyl-2-pyrrolidone) as a reducing agent. Nanoscale Res. Lett..

[22] Zulkifli ZA, Razak KA, Rahman WNWA, Abidin SZ (2018). Synthesis and characterisation of bismuth oxide nanoparticles using hydrothermal method: the effect of reactant concentrations and application in radiotherapy. J. Phys. Conf. Ser..

[23] Torrisi L, Silipigni L, Restuccia N, Cuzzocrea S, Cutroneo M, Barreca F, Fazio B, Di Marco G, Guglielmino S (2018). Laser-generated bismuth nanoparticles for applications in imaging and radiotherapy. J. Phys. Chem. Solids.

[24] Anandan S, Wu JJ (2009). Microwave Assisted Rapid Synthesis of Bi2O3 Short Nanorods. Mater. Lett..

[25] Mallahi M, Shokuhfar A, Vaezi MR, Esmaeilirad A, Mazinani V (2014). Synthesis and characterization of bismuth oxide nanoparticles via sol-gel method. AJER.

[26] Mädler L, Pratsinis SE (2004). Bismuth oxide nanoparticles by flame spray pyrolysis. J. Am. Ceram Soc..

[27] Carotenuto G, Hison CL, Capezzuto F, Palomba M (2009). Synthesis and thermoelectric characterisation of bismuth nanoparticles. J. Nanoparticle Res..

[28] Schulz S, Heimann S, Wölper C, Assenmacher W (2012). Synthesis of bismuth pseudocubes by thermal decomposition of Bi2Et4. Chem. Mater..

[29] Huang YJ, Zheng YQ, Zhu HL, Wang JJ (2016). Hydrothermal synthesis of bismuth (III) coordination polymer and its transformation to nano α-Bi2O3 for photocatalytic degradation. J. Solid State Chem..

[30] Gujar TP, Shinde VR, Lokhande CD, Mane RS, Han SH (2006). Formation of highly textured (111) Bi2O3 films by anodization of electrodeposited bismuth films. Appl. Surf. Sci..

[31] Gujar TP, Shinde VR, Lokhande CD (2008). The influence of oxidation temperature on structural, optical and electrical properties of thermally oxidized bismuth oxide films. Appl. Surf. Sci..

[32] Kim H, Jin C, Park S, Lee WI, Chin IJ, Lee C (2013). Structure and optical properties of Bi2S3 and Bi2O3 nanostructures synthesized via thermal evaporation and thermal oxidation routes. Chem. Eng. J..

[33] Nazari P, Faramarzi MA, Sepehrizadeh Z, Mofid MA, Bazaz RD, Shahverdi AR (2012). Biosynthesis of bismuth nanoparticles using Serratia marcescens isolated from the Caspian Sea and their characterization. IET Nanobiotechnol..

[34] Song JY, Kim BS (2009). Biological synthesis of bimetallic Au/Ag nanoparticles using Persimmon (Diospyros kaki) leaf extract. Korean J. Chem. Eng..

[35] Monda S, Roy N, Laskar RA, Sk I, Basu S, Mandal D, Begum NA (2011). Biogenic synthesis of Ag, Au and bimetallic Au/Ag alloy nanoparticles using aqueous extract of mahogany (Swietenia mahogani JACQ ) leaves. Colloid Surf. B..

[36] Dobrucka R (2017). Synthesis of titanium dioxide nanoparticles using Echinacea purpurea Herba. Iran. J. Pharm. Res..

[37] Aromal SA, Philip D (2012). Green synthesis of gold nanoparticles using Trigonella foenum-graecum and its size dependent catalytic activity. Spectrochim. Acta A..

[38] Tavakoli F, Salavati-Niasari M, Mohandes F (2015). Green synthesis and characterization of graphene nanosheets. Mater. Res. Bull..

[39] Kelkawi AHA, Abbasi Kajani A, Bordbar AK (2017). Green synthesis of silver nanoparticles using Mentha pulegium and investigation of their antibacterial, antifungal and anticancer activity. IET Nanobiotechnol..

[40] Jafari A, Pourakbar L, Farhadi K, Mohamadgolizad L, Goosta Y (2015). Biological synthesis of silver nanoparticles and evaluation of antibacterial and antifungal properties of silver and copper nanoparticles. Turk. J. Biol..

[41] Hajiashrafi S, Motakef-Kazemi N (2018). Green synthesis of zinc oxide nanoparticles using parsley extract. Nanomed. Res. J..

[42] Becheri A, Durr M, Nostro PL, Baglioni P (2008). Synthesis and characterization of zinc oxide nanoparticles: application to textiles as UV-absorbers. J. Nanopart. Res..

[43] Im YM, Oh TH, Nathanael JA, Jang SS (2015). Effect of ZnO nanoparticles morphology on UV blocking of poly (vinylalcohol)/ZnO composite nanofibers. Mater Lett..

[44] Bera KK, Majumdar M, Chakraborty M, Bhattachary SK (2018). Phase control synthesis of α, β and α/β Bi2O3 hetero-junction with enhanced and synergistic photocatalytic activity on degradation of toxic dye, Rhodamine-B under natural sunlight. J. Hazard. Mater..

